# Supplementary material for: Lattice oxygen-mediated electron tuning promotes electrochemical hydrogenation of acetonitrile on copper catalysts
Source: Nat Commun. 2023 Jun 29;14:3847. doi: 10.1038/s41467-023-39558-3 (PMC10310717; doi:10.1038/s41467-023-39558-3)
Supplement: Supplementary file 1 — Supplementary Information [file 41467_2023_39558_MOESM1_ESM.pdf]

Supplemental information for

Lattice oxygen mediated electron tuning promotes electrochemical hydrogenation of acetonitrile on copper catalysts

Cong Wei[1,†], Yanyan Fang[1,†], Bo Liu[1], Chongyang Tang[1], Bin Dong[2], Xuanwei Yin[1], Zenan Bian[1], Zhandong Wang[2], Jun Liu[3], Yitai Qian[1], Gongming Wang[1*]

[1] Department of Chemistry, University of Science and Technology of China, Hefei 230026, China

[2] National Synchrotron Radiation Laboratory, University of Science and Technology of China, Hefei 230029, China.

[3] Institute of Solid State Physics, Hefei Institutes of Physical Science, Chinese Academy of Sciences, Hefei 230031, China.

[†] These authors contributed equally: Cong Wei and Yanyan Fang

*Authors to whom any correspondence should be addressed

E-mail: wanggm@ustc.edu.cn;

Supplementary Methods

Supplementary Tables

Supplementary Table 1. Linear component fitting (LCF) dates of Cu and OD-Cu , respectively.

	E-space		
	Cu_foil	Cu_2O	R factor
OD-Cu NWs	0.72	0.28	0.0008
Cu NWs	1.00	0.00	0.0042

Supplementary Table 2. EXAFS fitting parameters at the Cu K-edge for various samples. (S_0^2=0.926)

Sample	Shell	^{a}N	^{b}R(Å)	$^{c}\sigma_2$(Å^2)	$^{d}\Delta E_0$	R factor
Cu Foil	Cu-Cu	12.0	2.54	0.0086	4.4	0.0029
CuO	Cu-O	3.72	1.94	0.0038	9.7	0.0140
Cu_2O	Cu-O	1.66	1.85	0.0028	8.3	0.0064
OD-Cu NWs	Cu-Cu	9.08	2.54	0.0088	5.0	0.0040
Cu NWs	Cu-Cu	10.25	2.54	0.0085	3.9	0.0033

^{a}N: coordination numbers; ^{b}R: bond distance; $^{c}\sigma_2$: Debye-Waller factors; $^{d}\Delta E_0$: the inner potential correction. R factor: goodness of fit. S_0^2 was set to 0.926, according to the experimental EXAFS fit of Cu foil reference by fixing coordination numbers as the known crystallographic value.

Supplementary Table 3. The table of performance comparison.

**Electrochemical reduction of acetonitrile to ethylamine**					
Catalyst	FE_{EA} (%)	Potential (V vs. RHE)	j_{total} (mA cm^{-2})	Electrolyte	Ref.
OD-Cu NWs	~92.3	0.16	30	Ar-saturated 1 M KOH 8 wt% AN	**This work**
	~97.8	-0.32	355		
	~96.05	-0.54	1442		
	~82	-	400 (Stability 16 h) MEA test		
Cu NWs	~72.7	-0.25	37		
	~93.4	-0.55	545		
	~81.2	-0.7	1191		
Cu nanoparticles	~94.6	-0.39	47.31	1 NaOH, 8wt% AN	Nat. Commun. 2021, 12, 1949.
	~86	-0.46	100 (Stability 20 h)		
	~55.7	-0.76	1000		
Ni	~80.56	-0.41	40.28		
	~53.59	-0.51	53.59		
	~16.00	-0.66	64.00		
Pd	~54.19	-0.40	27.10		
	~67.15	-0.49	67.15		
	~18.03	-0.65	72.11		
Cu NAs/Cu Foam	~41	−0.7 V (2h)	-	Ar-saturated 0.5 M $KHCO_3$ 0.5 M AN	Chem Catal., 2022, 2, 499-507.
	~94		-	CO_2-saturated 0.5 M $KHCO_3$ 0.5 M AN	
$[Co(DDP)(H_2O)_2](NO_3)_2$	~22	-1.87 V vs. Fc/Fc+	-0.02	Acetic acid	Dalton Trans. 2019, 48, 9576-9580.
$[Co(cis\text{-}DDOP)(NO_3)](NO_3)$	~11	−2.05 V vs. Fc/Fc+	-0.026		

Supplementary Figures

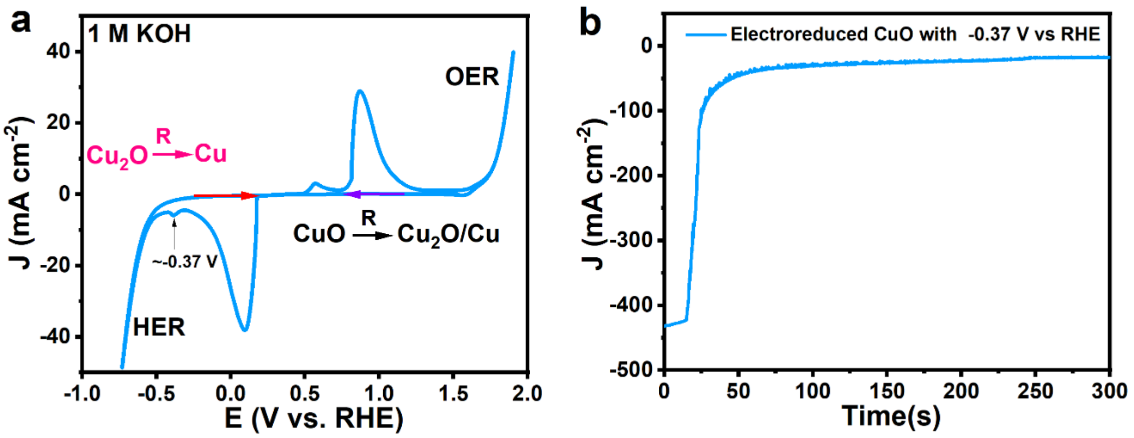

Supplementary Fig. 1 | *In situ* electrochemical synthesis of OD-Cu NWs. a, Cyclic voltammetry (CV) curve of CuO nanowires in 1.0 M KOH (R stands for reduction) at the scan rate of 10 mV s^{-1}. **b,** *In situ* synthesis of OD-Cu NWs by electroreduction at -0.37 V (vs RHE without iR correction).

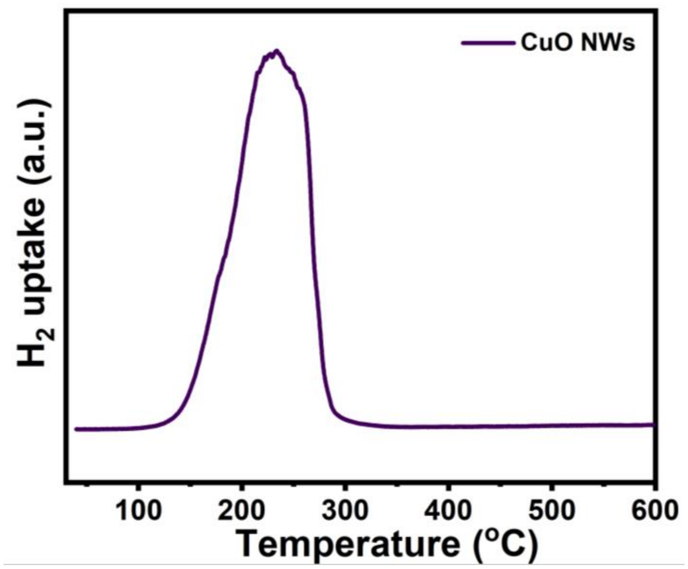

Supplementary Fig. 2 | H_2-TPR measurement of CuO NWs. The a.u. stands for arbitrary units.

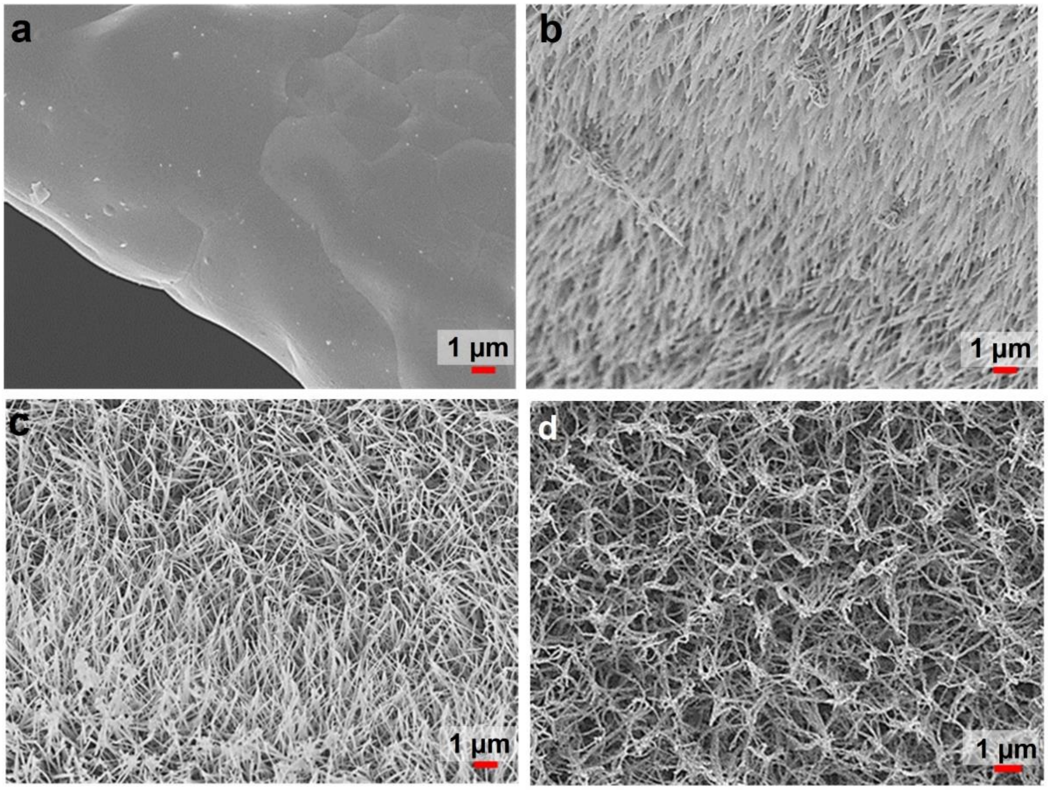

Supplementary Fig. 3 | SEM image of Cu catalysts. a, Cu Foil. **b**, $Cu(OH)_2$. **c**, CuO. **d**, Cu NWs.

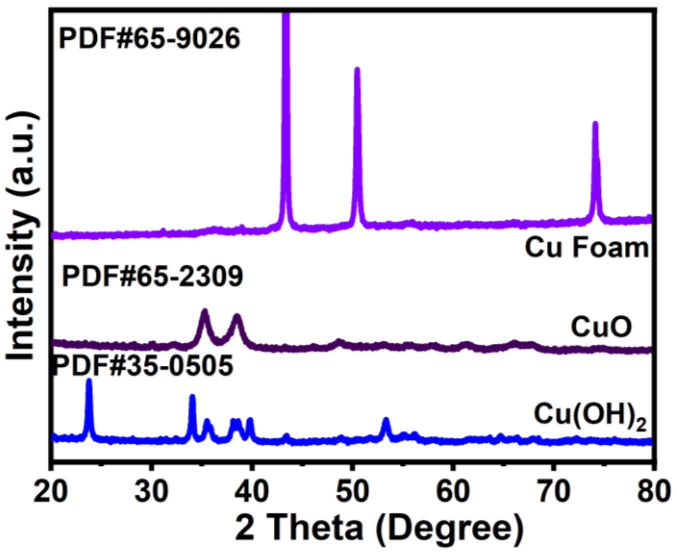

Supplementary Fig. 4 | The XRD profiles of Cu Foam, CuO, and Cu(OH)$_2$. The a.u. stands for arbitrary units.

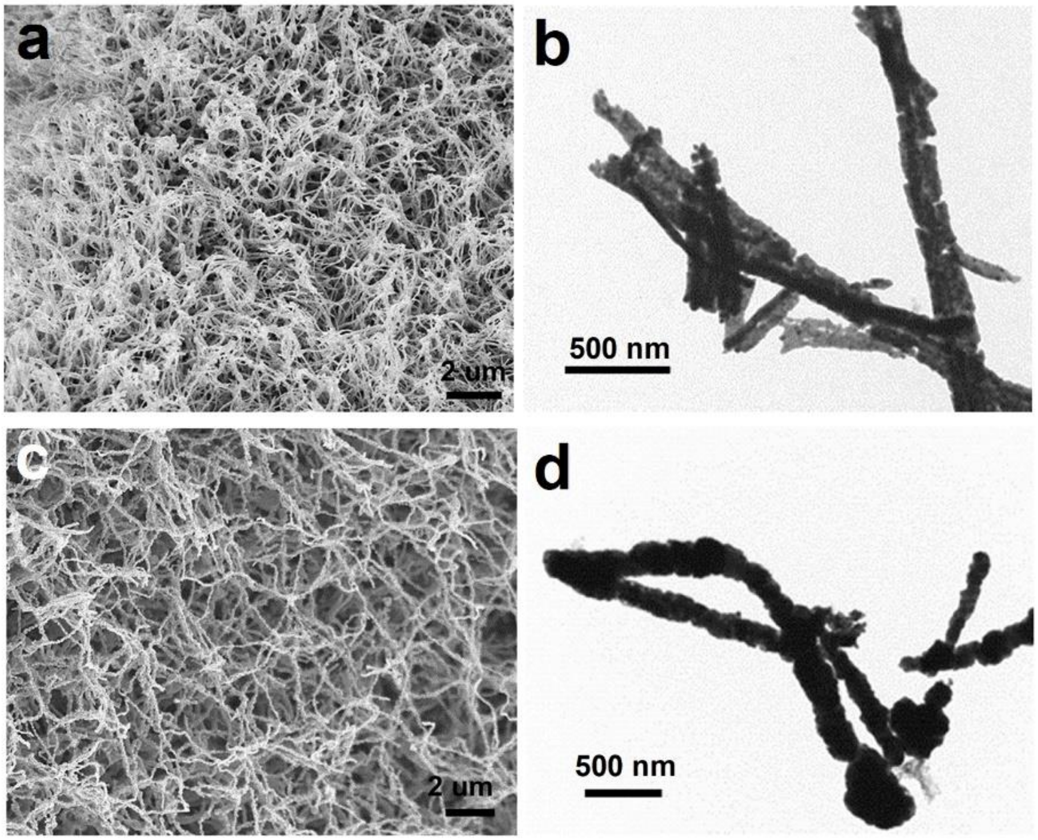

Supplementary Fig. 5 | SEM and TEM images of Cu catalysts. a,b OD-Cu NWs and **c,d** Cu NWs.

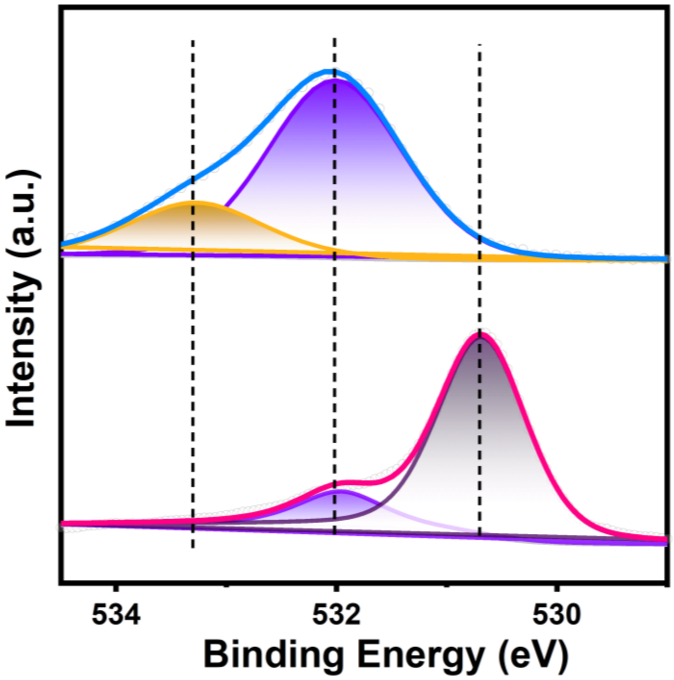

Supplementary Fig. 6 | O 1s XPS spectra of Cu NWs and OD-Cu NWs. The a.u. stands for arbitrary units.

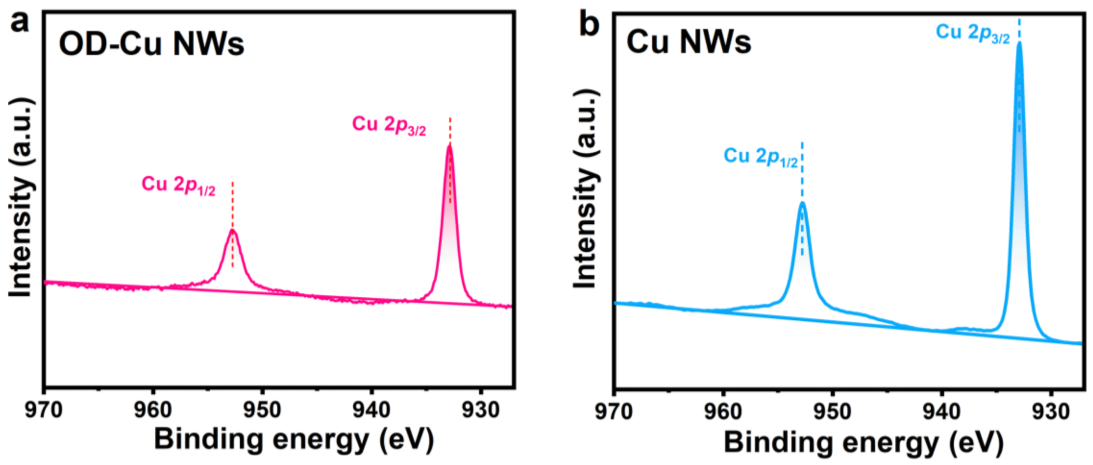

Supplementary Fig. 7 | Cu 2p XPS spectra of Cu catalysts. a, OD-Cu NWs. **b,** Cu NWs. The a.u. stands for arbitrary units.

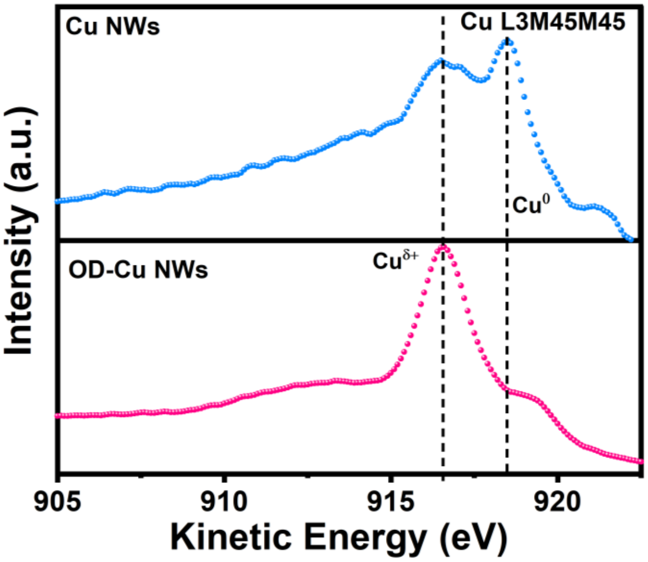

Supplementary Fig. 8 | L3M45M45 Auger spectra of Cu NWs(blue) and OD-Cu NWs (red). The a.u. stands for arbitrary units.

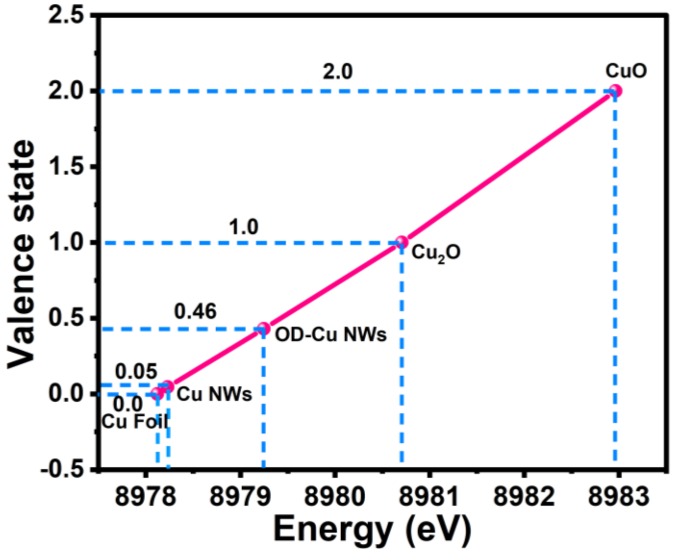

Supplementary Fig. 9 | The detailed valence states of Cu catalysts.

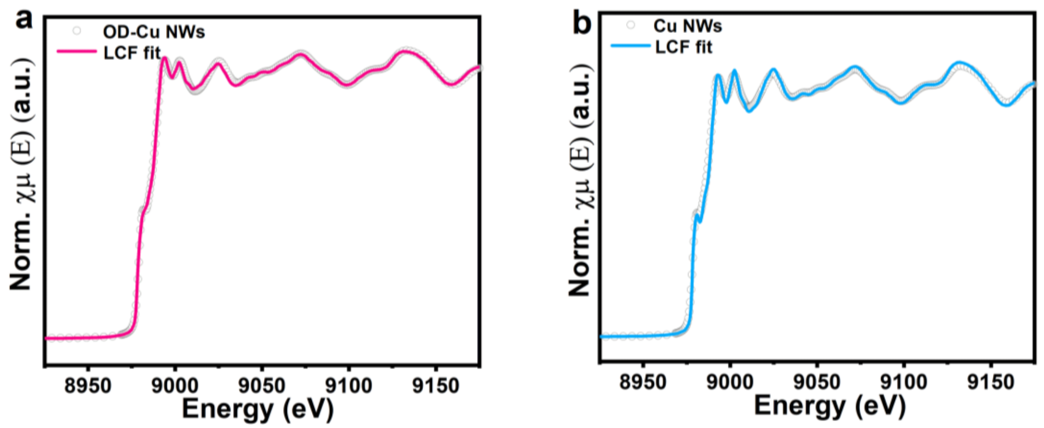

Supplementary Fig. 10 | Linear component fitting (LCF) of k3-weighted XAFS spectra of Cu catalysts. **a**, OD-Cu NWs. **b**, Cu NWs. The a.u. stands for arbitrary units.

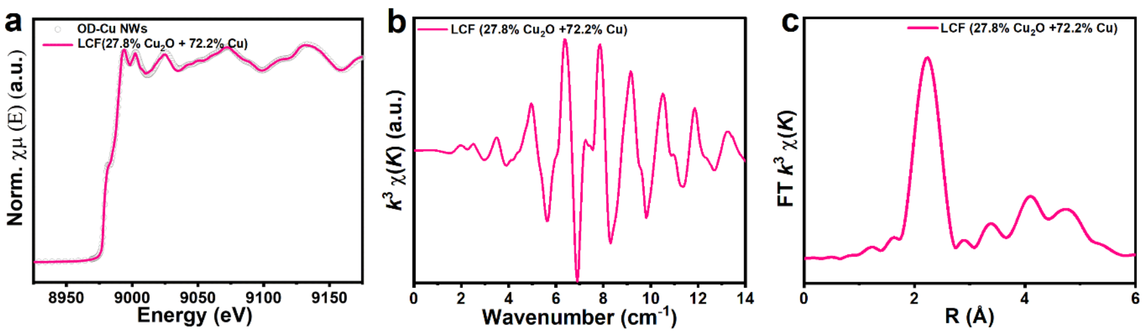

Supplementary Fig. 11 | XANES and EXAFS analysis of OD-Cu NWs. a, Cu K-edge linear component fit (LCF) XANES spectra. **b,** k^3-weighted EXAFS spectra and **c**, corresponding FT-EXAFS spectra. The a.u. stands for arbitrary units.

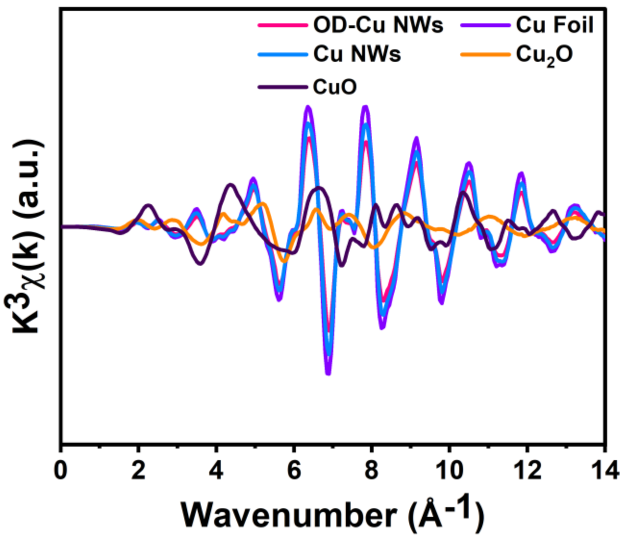

Supplementary Fig. 12 | The k3-weighted EXAFS spectra of Cu Foil, Cu NWs, OD-Cu NWs, Cu_2O and CuO. The a.u. stands for arbitrary units.

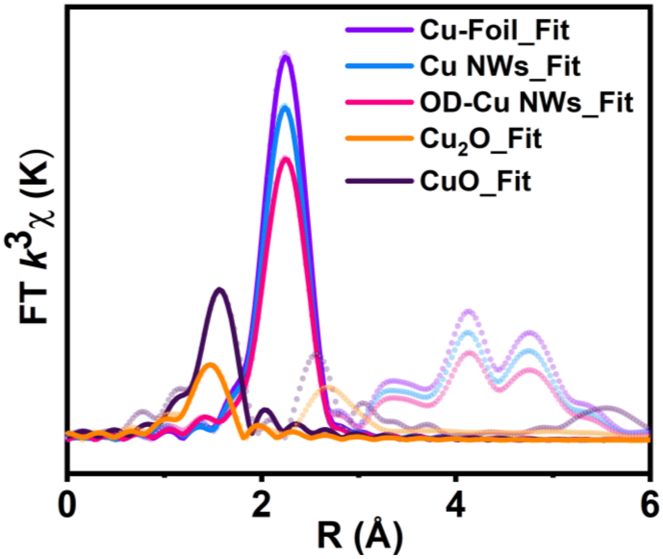

Supplementary Fig. 13 | The Fourier transformed (FT) k3-weighted EXAFS spectra of Cu foil, OD-Cu NWs, Cu NWs, Cu_2O and CuO.

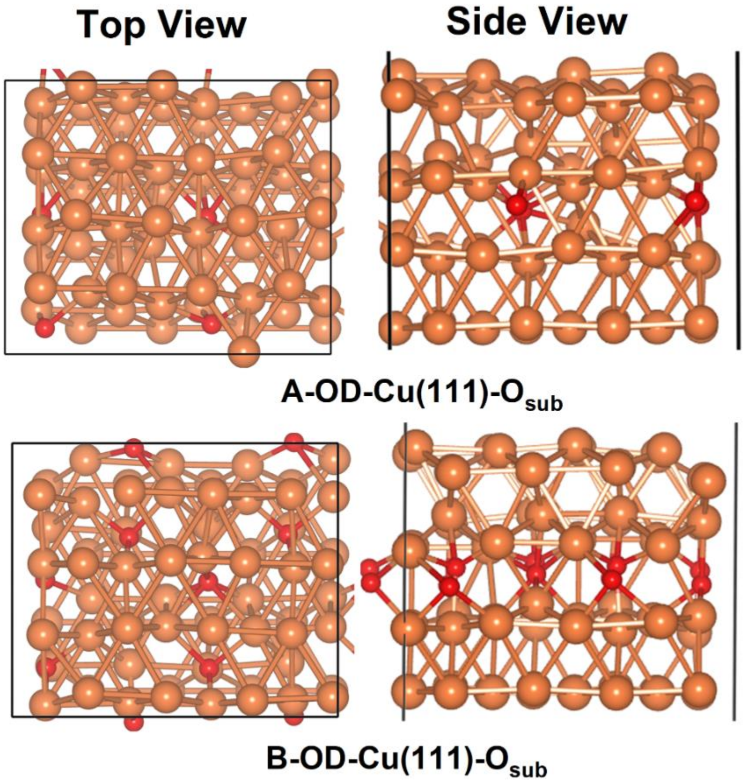

Supplementary Fig. 14 | The configuration of OD-Cu catalysts containing different lattice oxygen contents (A-OD-Cu(111)-O_{sub} and B-OD-Cu(111)-O_{sub}) after 300 K, 30 ps AIMD simulation.

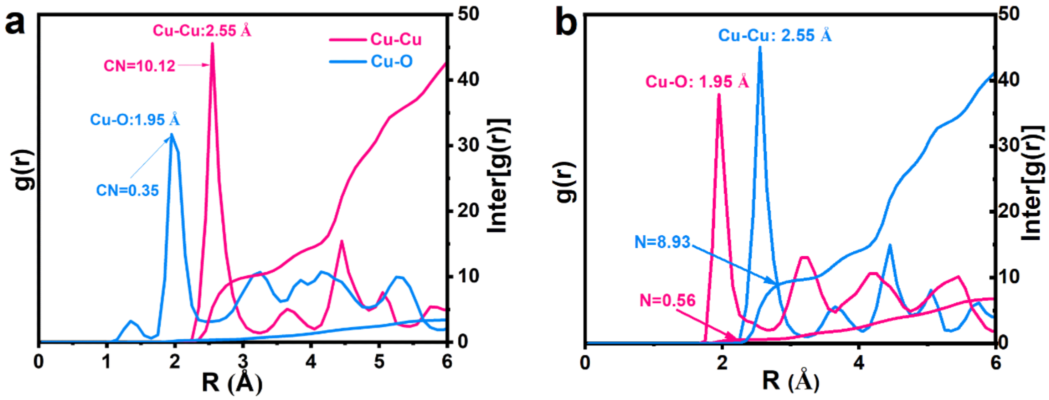

Supplementary Fig. 15 | AIMD simulation of RDFs between Cu-Cu and Cu-O on **a,** A-OD-Cu (111) and **b,** B-OD-Cu (111) surfaces.

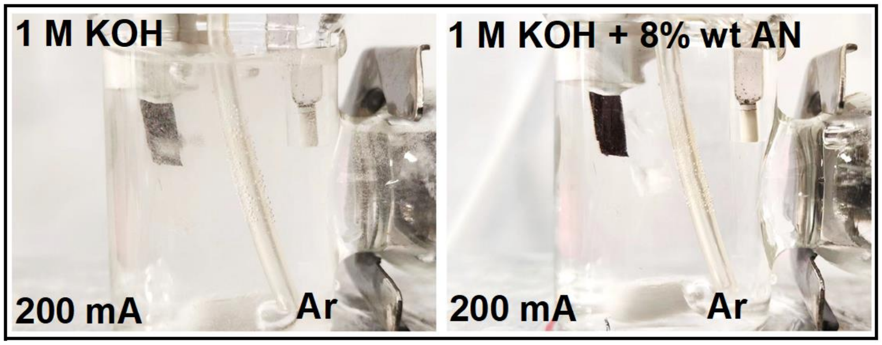

Supplementary Fig. 16 | Optical photographs of the electrochemical acetonitrile hydrogenation process on OD-Cu NWs. In the blank KOH solution (left), HER reaction occurs, and when acetonitrile is added to the solution (right), the HER reaction is significantly suppressed.

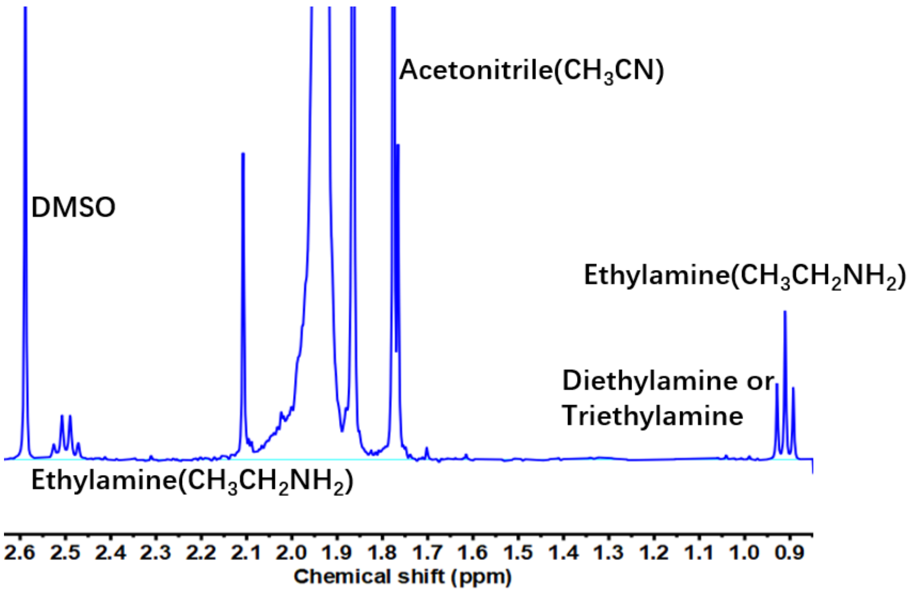

Supplementary Fig. 17 | The ^{1}H NMR spectrums of the liquid products obtained upon CH_3CN reduction on OD-Cu NWs at the applied potentials of -1.6 V versus SHE for 600 s. DMSO was used as an internal standard for quantification of liquid products.

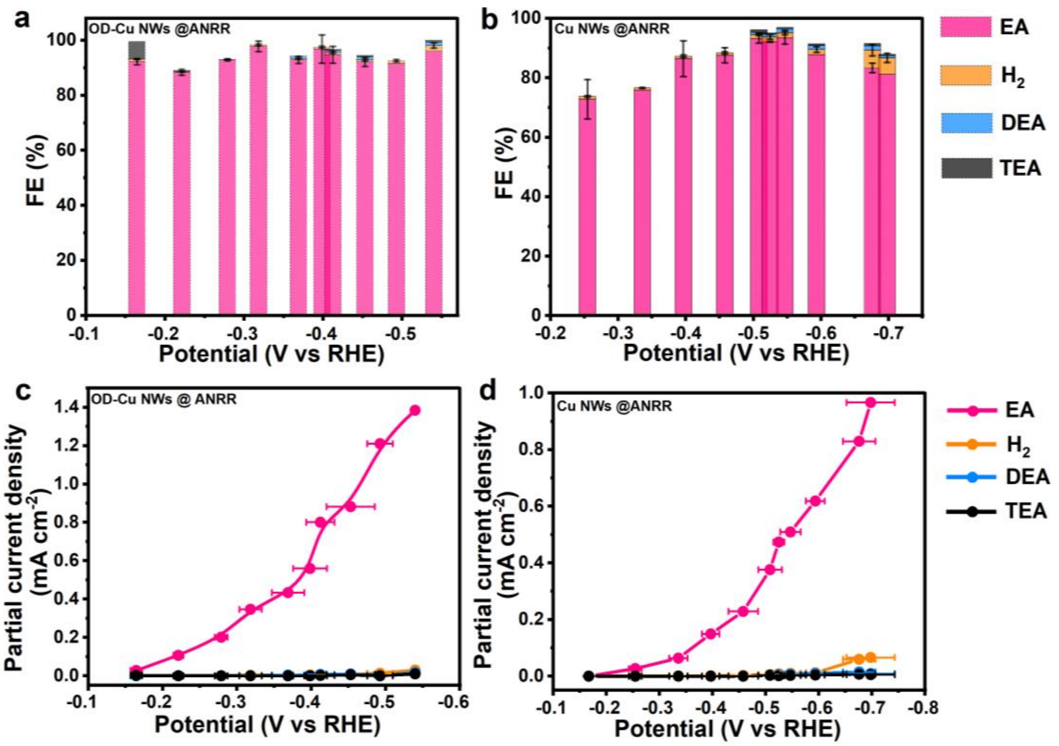

Supplementary Fig. 18 | Faraday efficiencies and partial current densities of various reduction products at different potentials on **a,c** OD-Cu NWs and **b,d** Cu NWs.

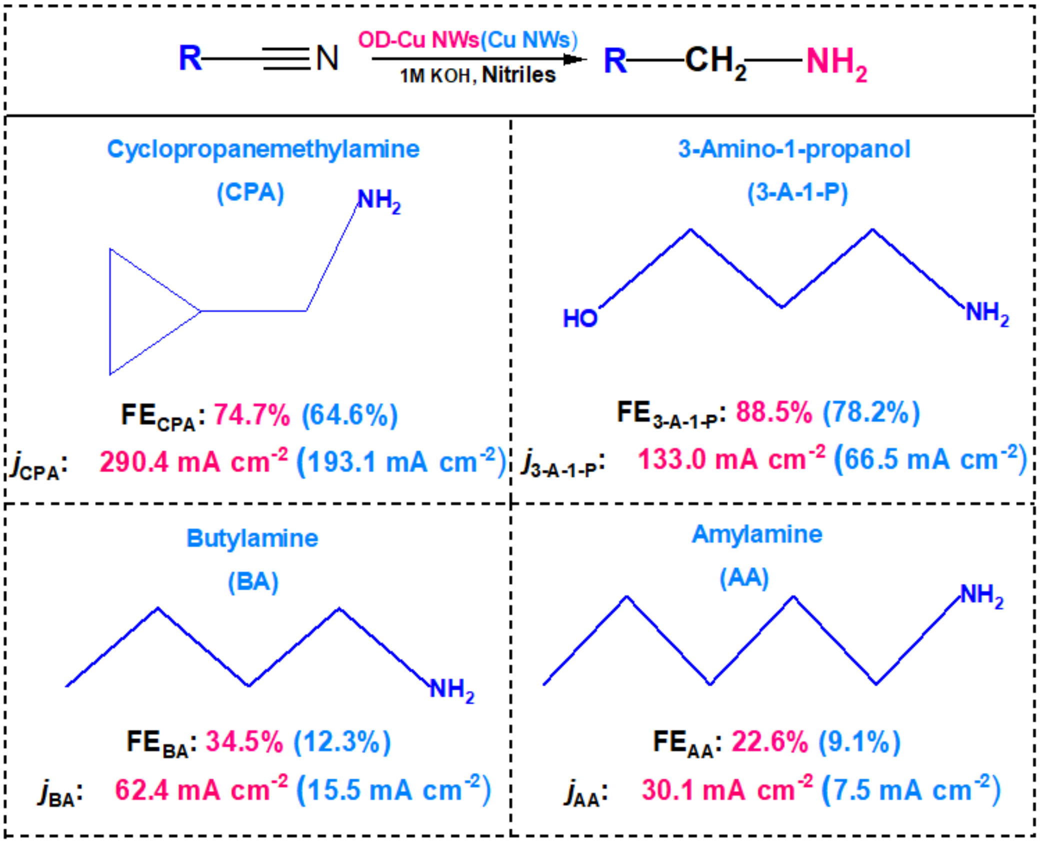

Supplementary Fig. 19 | Electrochemical hydrogenation of nitriles (Cyclopropanecarbonitrile (CPN), 3-Hydroxypropionitrile (3-HPN), Butyronitrile (BN), and Pentanenitrile (PN)).

The hydrogenation of other nitrile was further explored, including Cyclopropanecarbonitrile (CPN), 3-Hydroxypropionitrile (3-HPN), Butyronitrile (BN), and Pentanenitrile (PN), as shown in **Supplementary Figs. 20-23**. For all the studied small molecular nitriles, the conversions to amines on OD-Cu NWs are always better than on Cu NWs, with both higher current densities and Faradic efficiencies, which further demonstrates residual oxygen in OD-Cu NWs can efficiently manipulate the catalytic reduction of nitriles to primary amines. For the hydrogenation of butyronitrile (BN) and pentanenitrile (PN), the lower FEs of 34.5% and 22.6% are mainly due to their lower solubility.

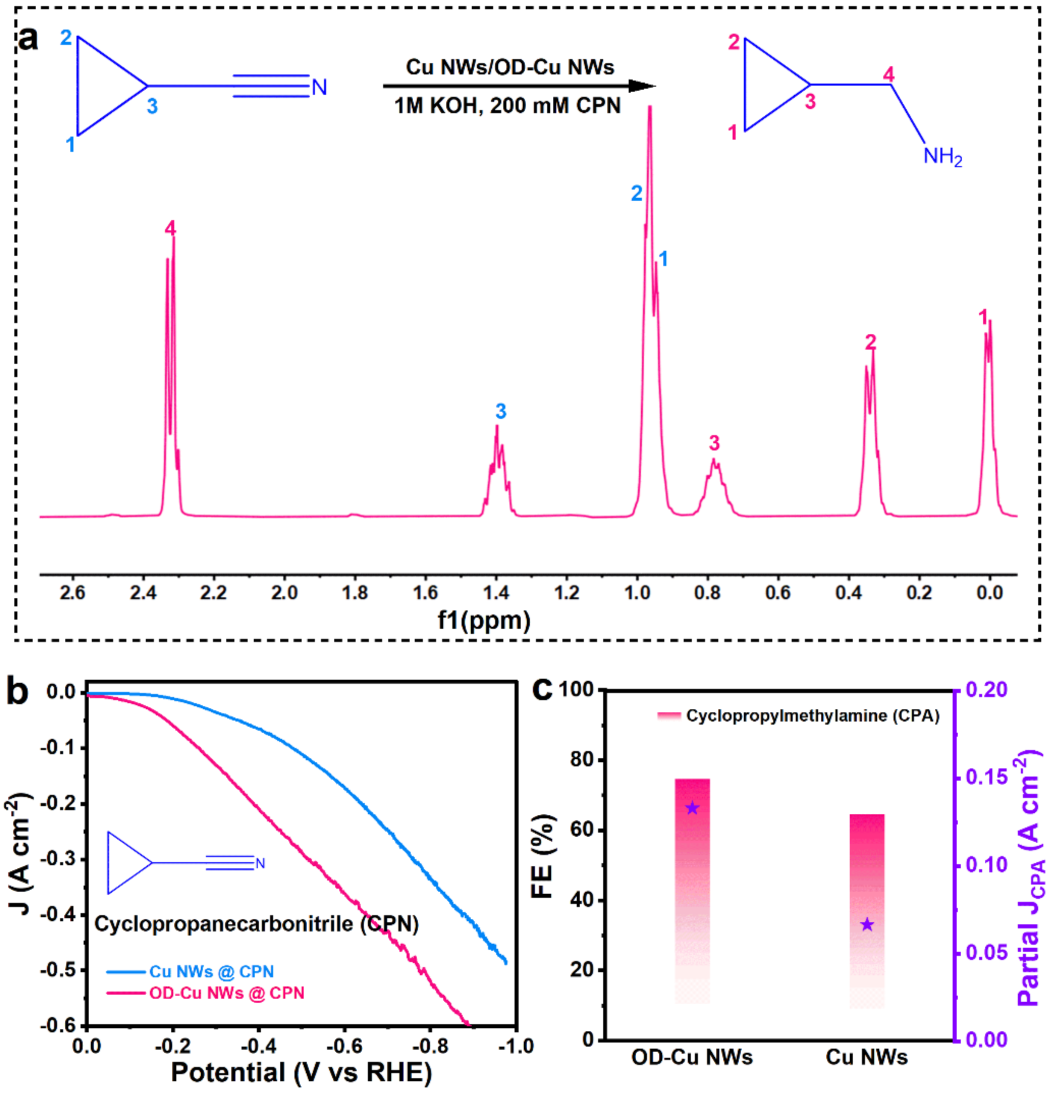

Supplementary Fig. 20 | Electrochemical hydrogenation of cyclopropanecarbonitrile (CPN). a, The ^{1}H NMR spectrums of CPN and CPA. **b**, LSV plots of Cu NWs and OD-Cu NWs in argon saturated 1 M KOH aqueous solutions with the addition of CPN (200 mM)). The scan rate was 10 mV s^{-1}. **c**, FE_{CPA} and the corresponding partial current densities at optimal electrolytic potential on Cu NWs and OD-Cu NWs.

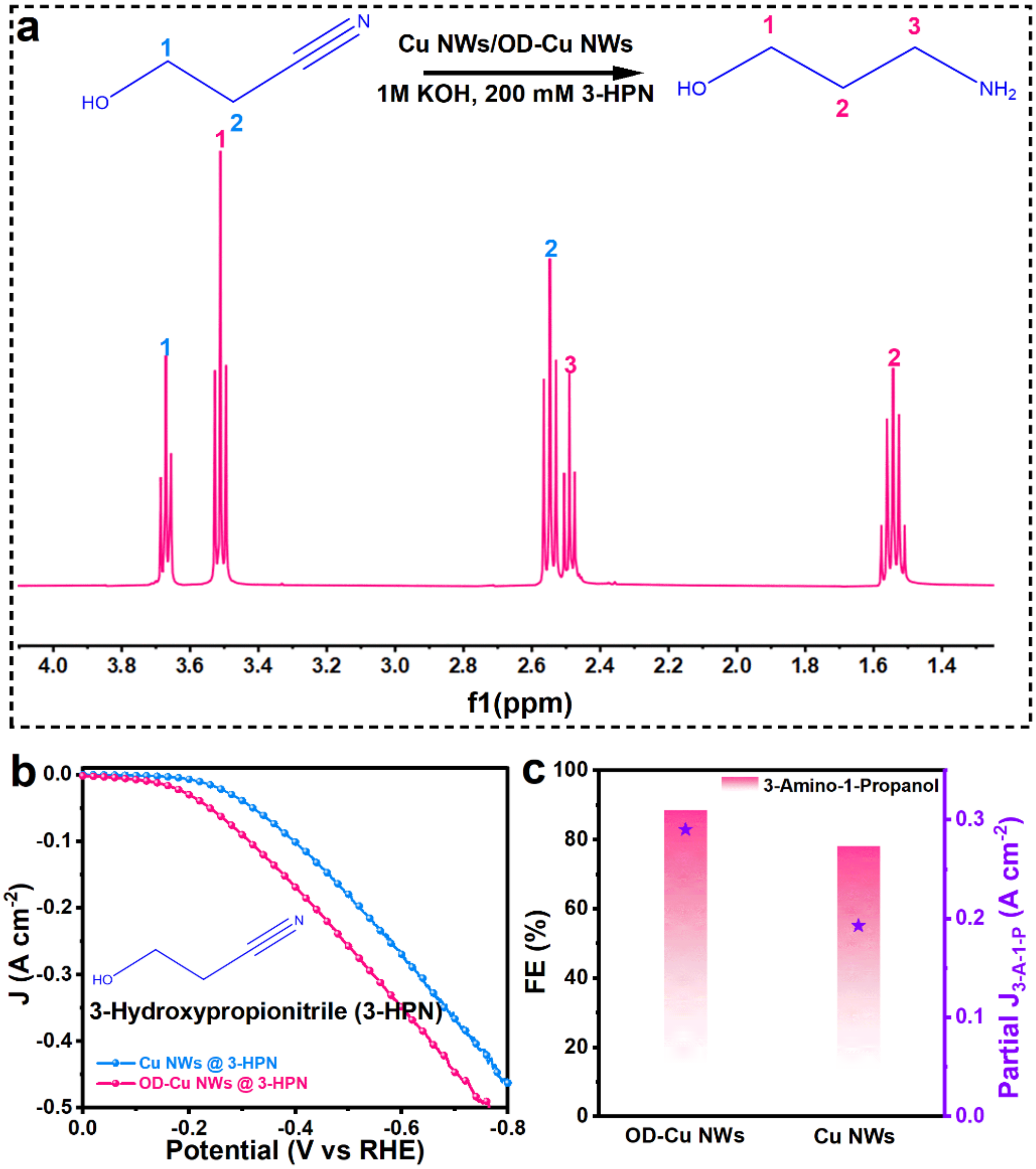

Supplementary Fig. 21 | Electrochemical hydrogenation of 3-Hydroxypropionitrile (3-HPN). a, The ^{1}H NMR spectrums of 3-HPN and 3-Amino-1-Propanol. **b**, LSV plots of Cu NWs and OD-Cu NWs in argon saturated 1 M KOH aqueous solutions with the addition of 3-HPN (200 mM). The scan rate was 10 mV s^{-1}. **c**, $FE_{3\text{-Amino-1-Propanol}}$ and the corresponding partial current densities at optimal electrolytic potential on Cu NWs and OD-Cu NWs.

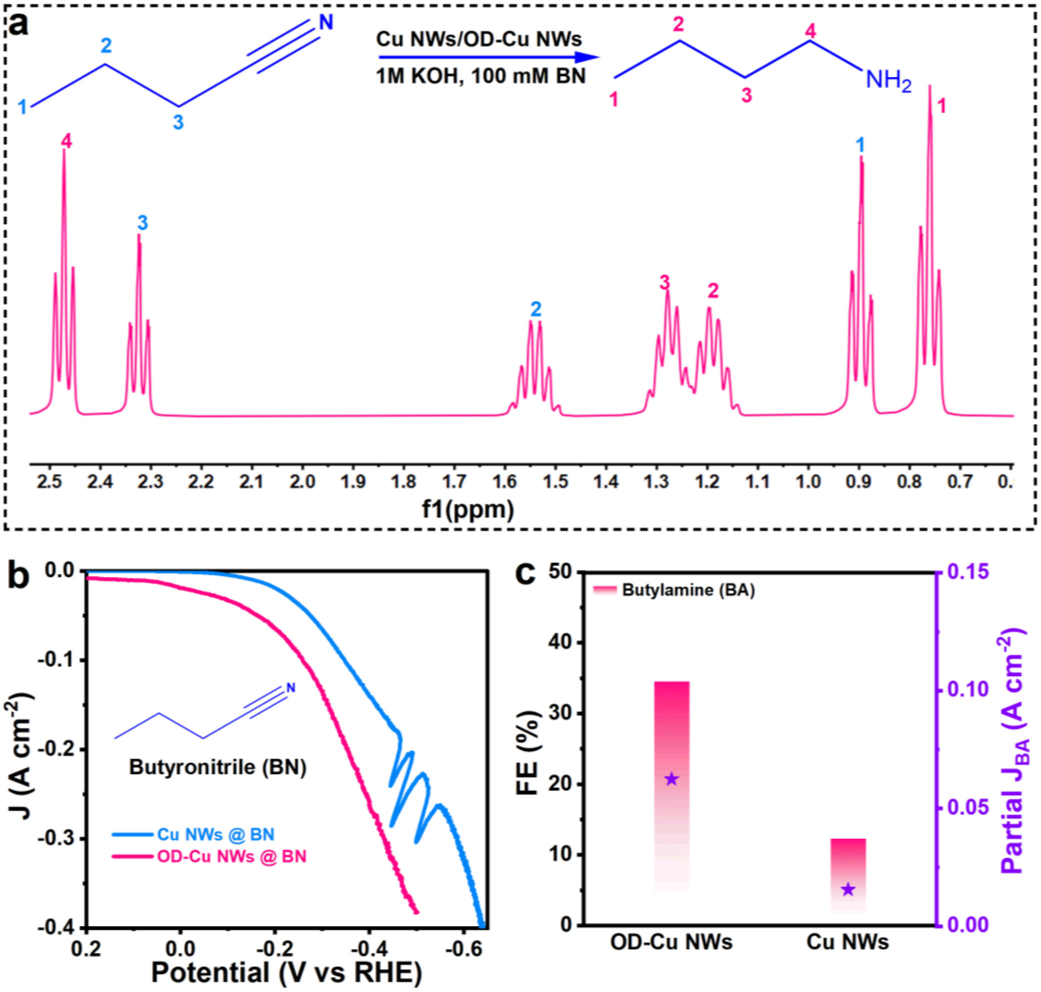

Supplementary Fig. 22 | Electrochemical hydrogenation of Butyronitrile (BN). a, The ^{1}H NMR spectrums of BN and BA. **b**, LSV plots of Cu NWs and OD-Cu NWs in argon saturated 1 M KOH aqueous solutions with the addition of BN (100 mM)). The scan rate was 10 mV s^{-1}. **c**, FE_{BA} and the corresponding partial current densities at optimal electrolytic potential on Cu NWs and OD-Cu NWs.

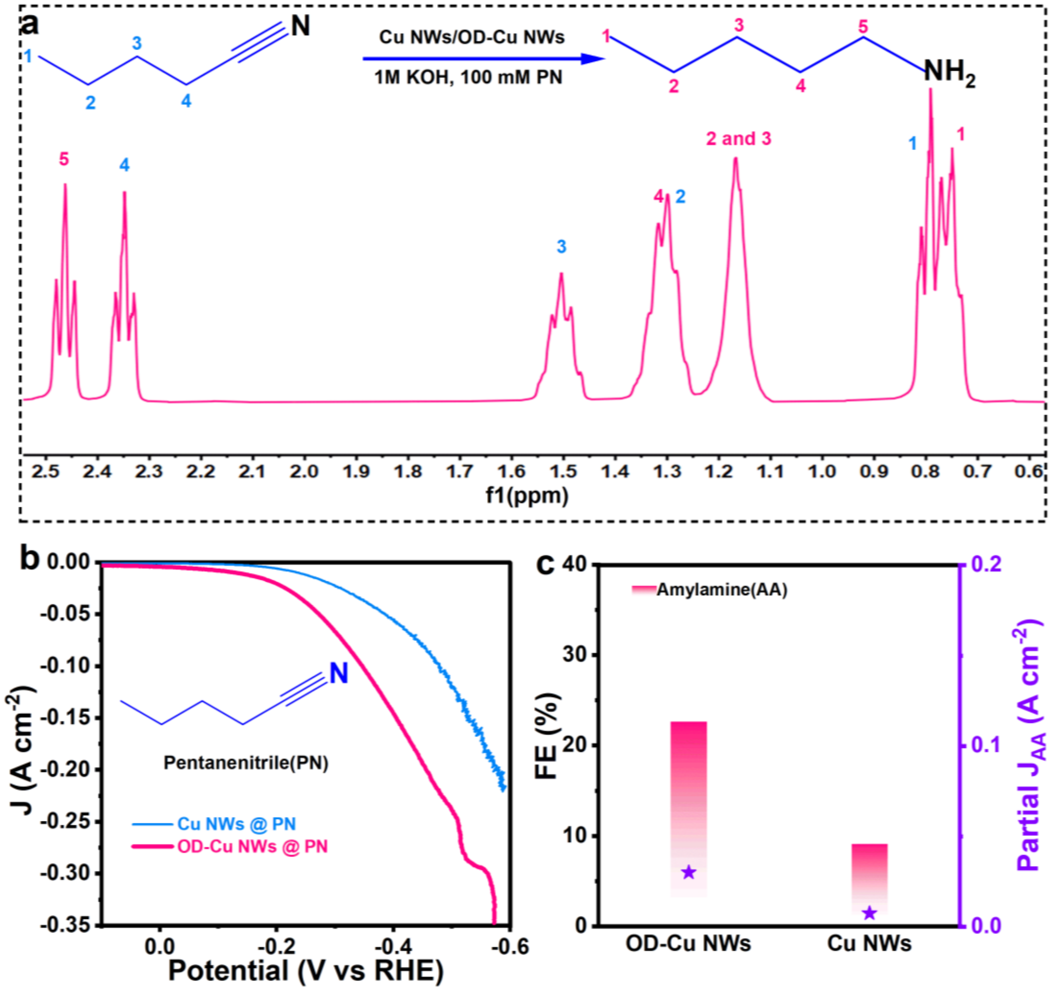

Supplementary Fig. 23 | Electrochemical hydrogenation of Pentanenitrile (PN)). **a,** The ^{1}H NMR spectrums of PN and AA. **b**, LSV plots of Cu NWs and OD-Cu NWs in argon saturated 1 M KOH aqueous solutions with the addition of PN (100 mM)). The scan rate was 10 mV s^{-1}. **c**, FE_{AA} and the corresponding partial current densities at optimal electrolytic potential on Cu NWs and OD-Cu NWs.

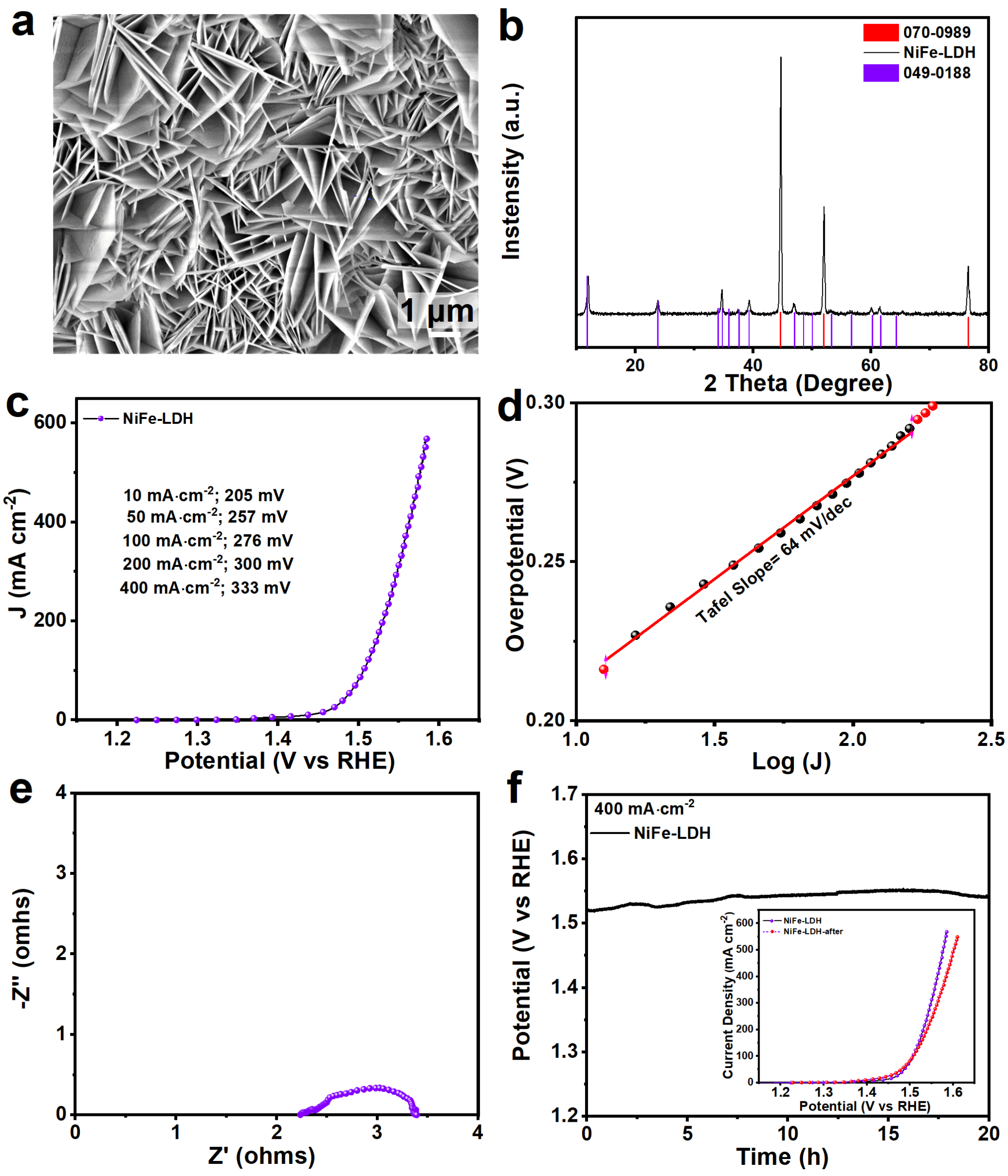

Supplementary Fig. 24 | Catalytic performances of NiFe LDH sample in the oxygen evolution reaction (OER). a, SEM image and **b,** XRD of NiFe LDH. **c**, The LSV curve of NiFe LDH catalyst (Scan rate: 5 mV s^{-1}) and **d**, the corresponding Tafel slope. **e,** Nyquist plots of NiFe LDH at the overpotential of 100 mV. **f**, The chronopotentiometry curve of NiFe LDH at 400 mA cm^{-2} for 20 h. The inset shows the LSV curves before and after the stability test. The a.u. stands for arbitrary units.

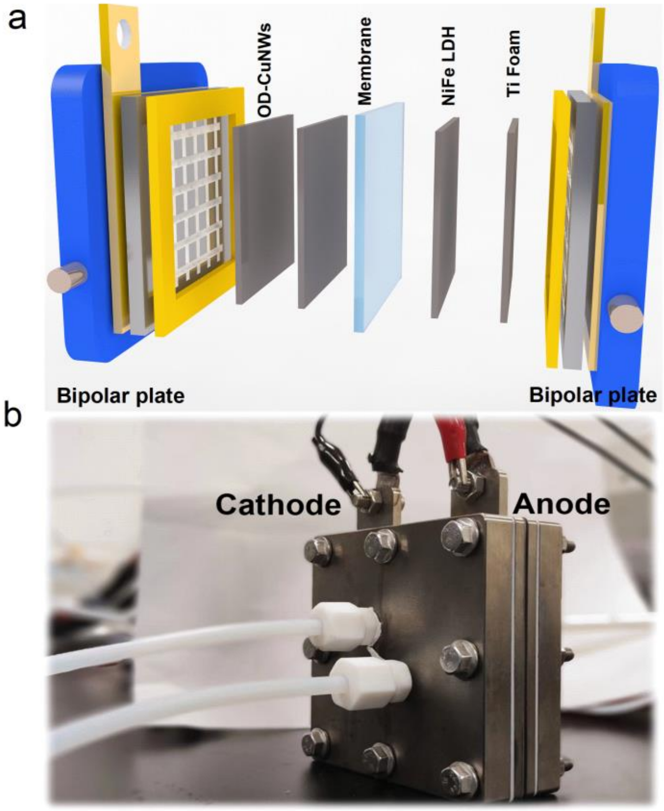

Supplementary Fig. 25 | Anion-exchange membrane-membrane electrode assembly (AEM-MEA) test. a, The schematic diagram of the test devices. **b**, Optical photograph of the AEM-MEA device.

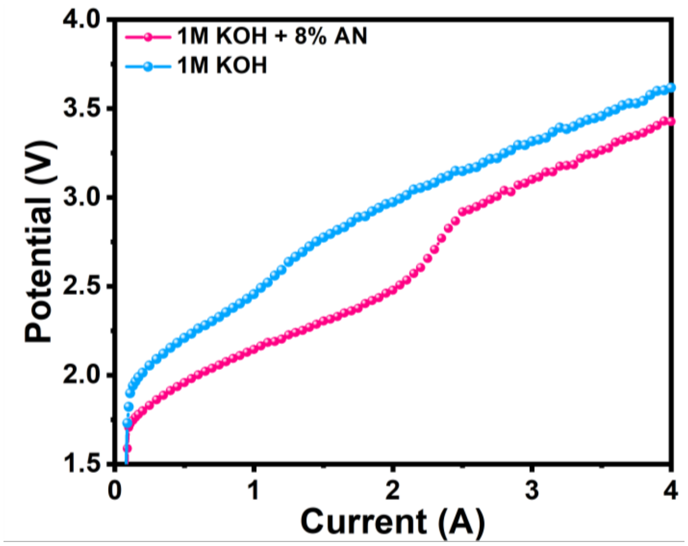

Supplementary Fig. 26 | V-I plots of OD-Cu NWs tested using AEM-MEA in 1M KOH argon saturated aqueous solution with and without the addition of AN (8 wt%).

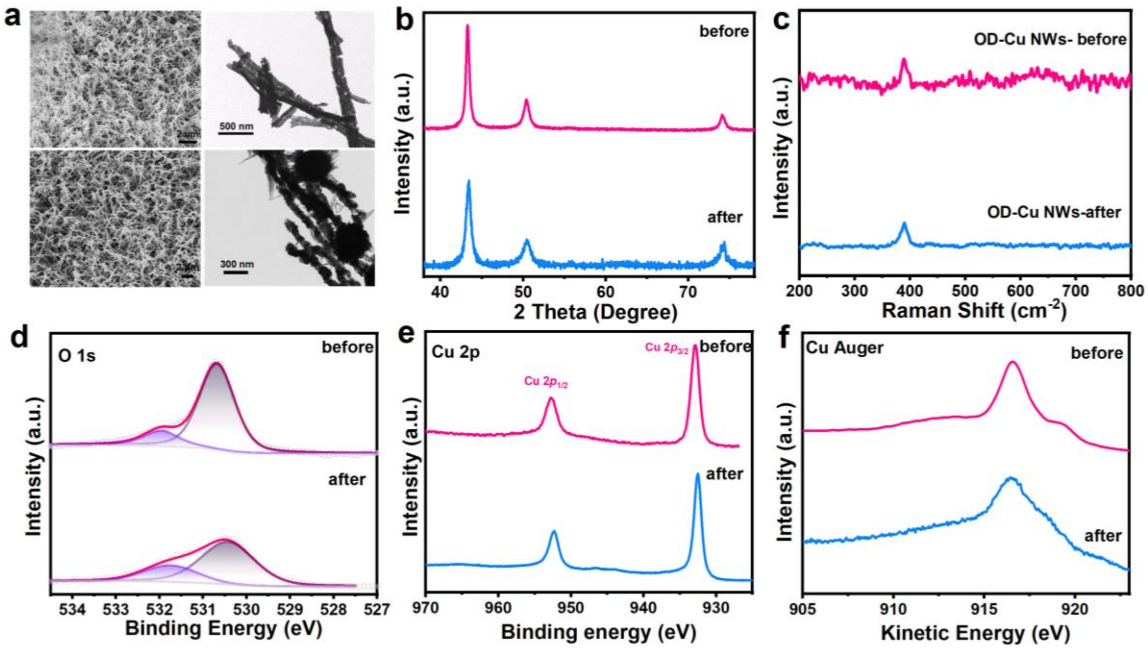

Supplementary Fig. 27 | Post-test characteristics of structure and valence. a, SEM and TEM images. **b**, XRD patterns. **c**, Raman spectra. XPS spectra of **d,** O 1s, **e,** Cu 2p and **f,** corresponding Auger spectra of OD-Cu NWs before and after the test. The a.u. stands for arbitrary units.

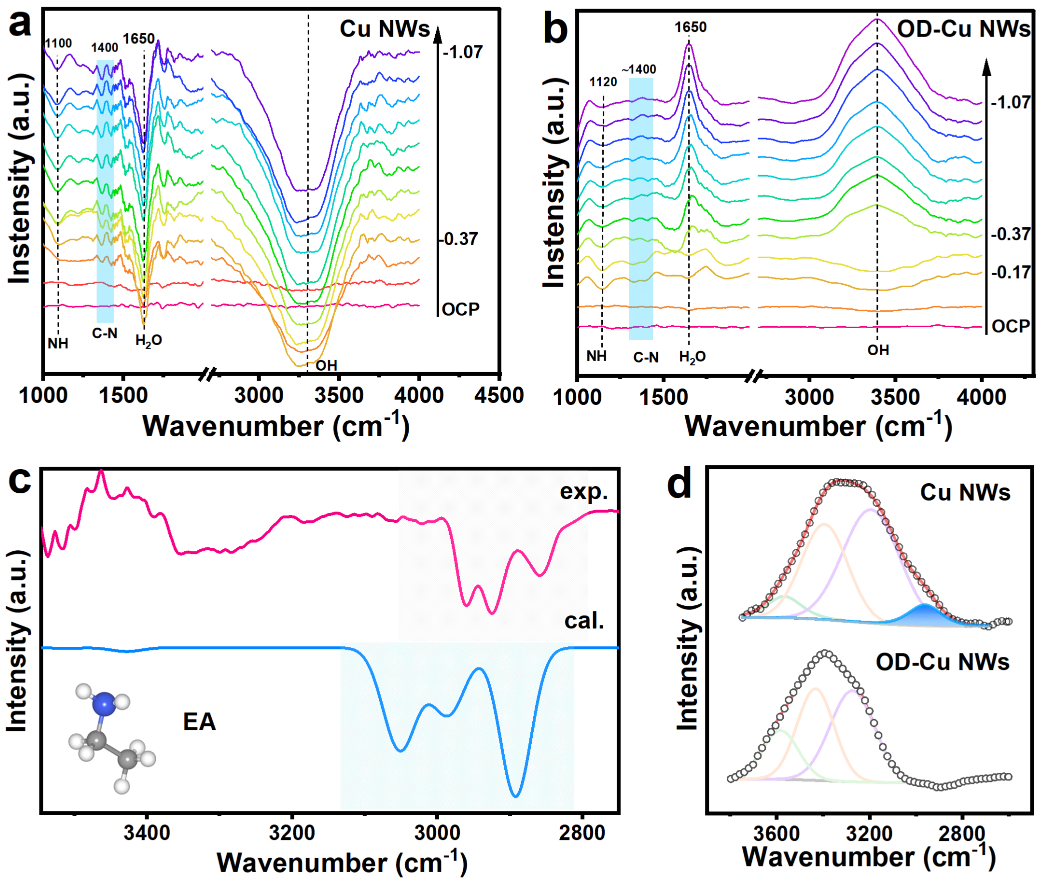

Supplementary Fig. 28 | *In situ* SR-FTIR characterization of the ANRR mechanism. 2D plots of *in situ* SR-FTIR spectra of **a,** Cu NWs and **b**, OD-Cu NWs at different potentials. **c,** Experimental and theoretical simulations of infrared spectra of ethylamine solutions. **d**, Peak fits of Cu NWs and OD-Cu NWs in the 2800-3750 cm^{-1} SR-FTIR spectral region at -1.07 V vs. RHE (without iR compensation). The a.u. stands for arbitrary units.

To distinguish the contribution of *v*(NH_2) vibration and the *v*(OH) vibration, Gaussian deconvolution method has been used to fit the IR spectrum[1]. According to the experimental data and theoretical simulations of EA, the *v*(NH_2) exists at approximately 3000 cm^{-1} (**Supplementary Fig. 23 c**). Furthermore, the experimental signal within 2800-3750 cm^{-1} are quantitatively fitted in the region of 2800-3750 cm^{-1}, where the presence of NH_2 is observed on Cu NWs but not exists on OD-Cu NWs (**Supplementary Fig. 23 d**).

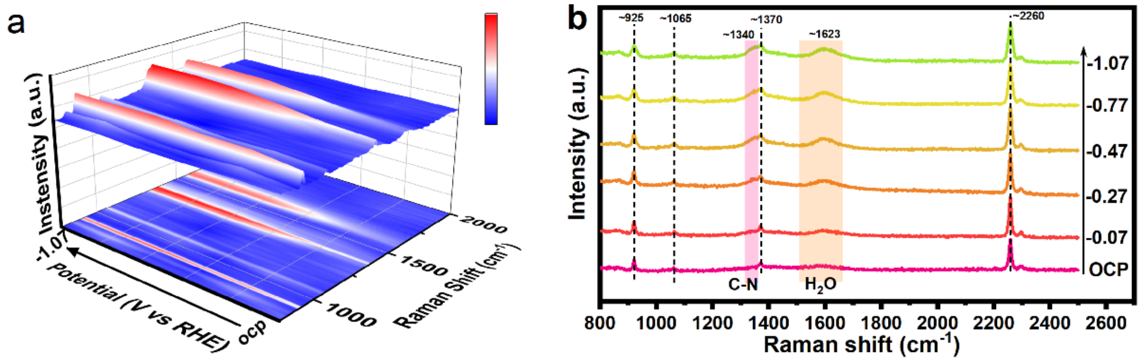

Supplementary Fig. 29 | *In situ* Raman characterization of the ANRR mechanism. a, 3D and **b**, 2D plots of in situ Raman spectra of OD-Cu NWs at different potentials. The a.u. stands for arbitrary units.

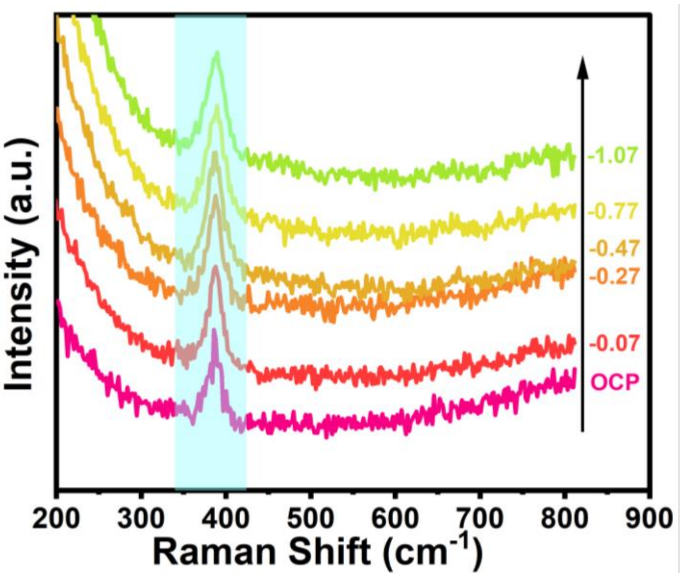

Supplementary Fig. 30 | *In situ* Raman spectra of OD-Cu NWs at different potentials. The a.u. stands for arbitrary units.

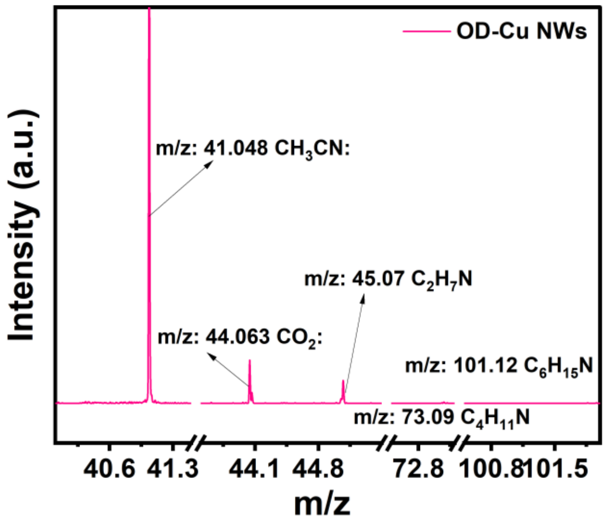

Supplementary Fig. 31 | The SVUV-PIMS signal at the ionization energy 11.6 eV. The a.u. stands for arbitrary units.

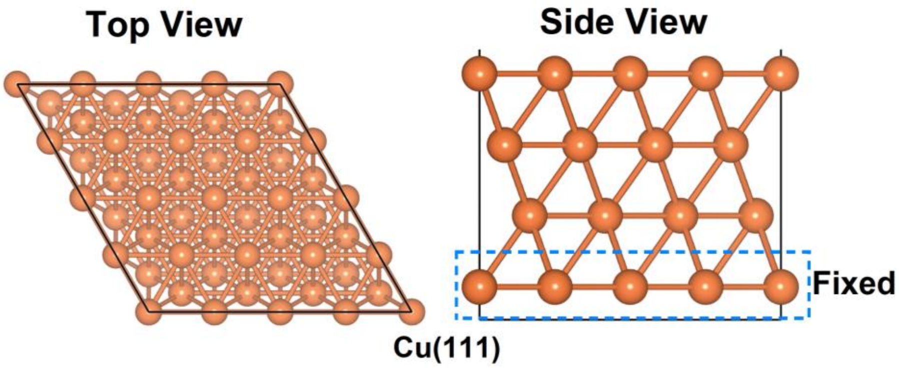

Supplementary Fig. 32 | Surface model of Cu(111).

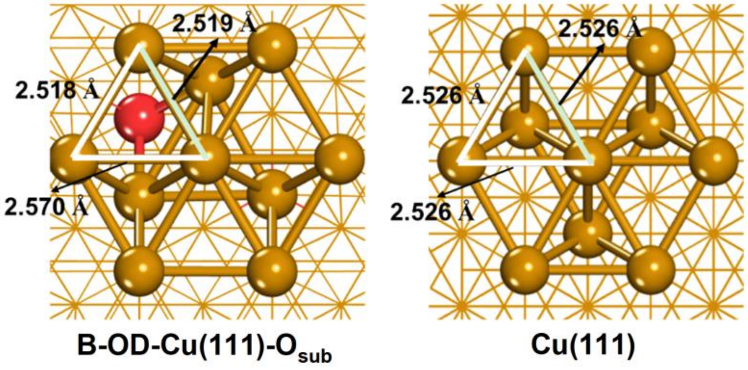

Supplementary Fig. 33 | Cu-Cu bond lengths of B-OD-Cu(111)-O_{sub} and Cu(111).

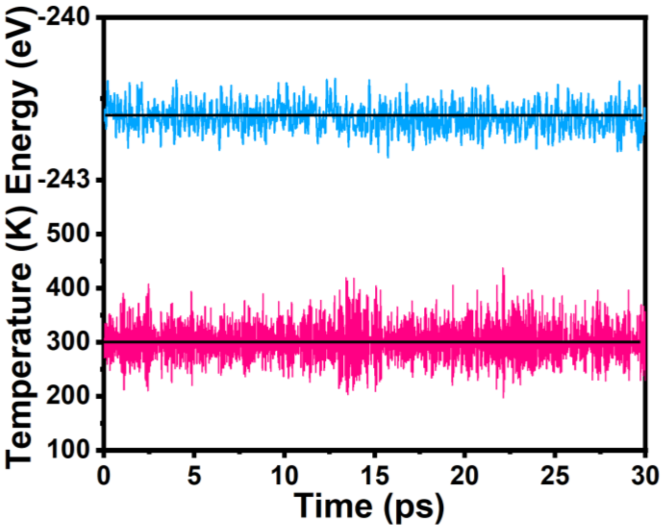

Supplementary Fig. 34 | A selected paradigm of temperature and potential energy evolution during AIMD simulations of B-OD-Cu (111)-O_{sub} at 300 K.

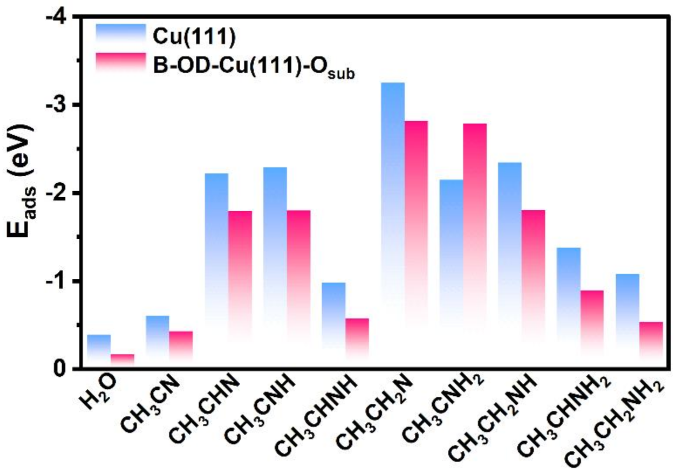

Supplementary Fig. 35 | The adsorption energies of the key intermediates involved in acetonitrile reduction reaction to form ethylamine are compared among the Cu (111), and B-OD-Cu (111)-O$_{sub}$ surfaces.

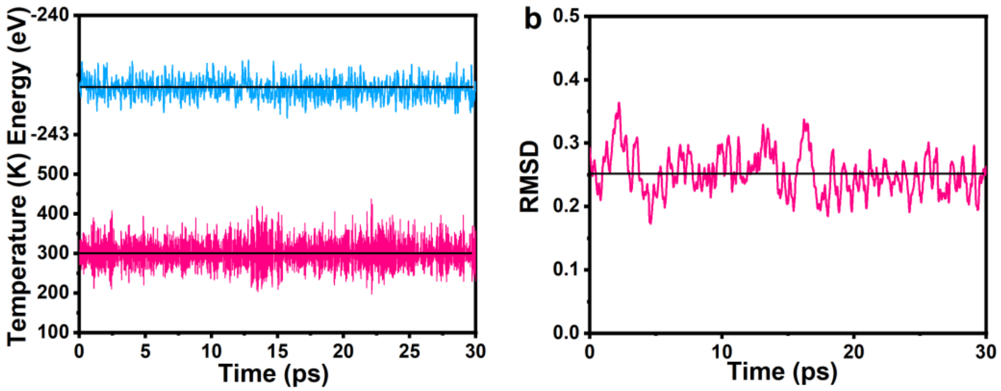

Supplementary Fig. 36 | AIMD simulations. a, A selected paradigm of temperature and potential energy evolution during AIMD simulations of A-OD-Cu(111)-O_{sub} at 300 K. **b**, Root-mean-square deviation (RMSD) of atom positions of A-OD-Cu(111)-O_{sub} surface in AIMD simulations at 300 K.

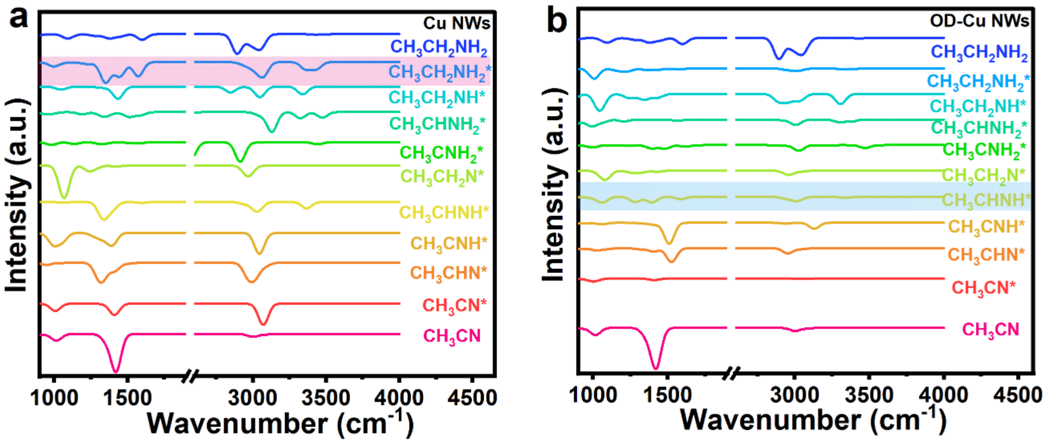

Supplementary Fig. 37 | Infrared spectral (IR) simulation. IR spectra of intermediates involved in acetonitrile electroreduction on **a**, Cu(111) and **b**, B-OD-Cu (111). The a.u. stands for arbitrary units.

The IR spectra of all intermediate species involved in the electroreduction of acetonitrile to ethylamine in their most stable adsorption configurations on Cu(111) and B-OD-Cu(111) surfaces are further investigated using the symmetry-based DFTP method (**Supplementary Fig. 32 a,b**). The results are compared with the experimental in-situ SR-FTIR spectra (**Supplementary Fig. 23 a,b**). Among the molecules considered in the calculation, the computational IR spectrum of ethylamine adsorbed on the Cu (111) surface (EA*) matches well with the in-situ SR-FTIR results obtained on Cu NWs throughout the potential region during the test. It can be inferred that the desorption of EA* is the potential determining step (PDS) on Cu(111) surface in the reaction due to its strong adsorption. In contrast, based on the calculated IR spectra on B-OD-Cu(111) surface, the signal of CH_3CHNH^* is consistent with the experimental SR-FTIR results from OD-Cu NWs within the testing potential range. Thus, on B-OD-Cu(111), the desorption of EA could be more favorable than on Cu(111), and the PDS is shifted to the proton-coupled electron transfer electrochemical process of CH_3CHNH^* to $CH_3CH_2NH^*$.

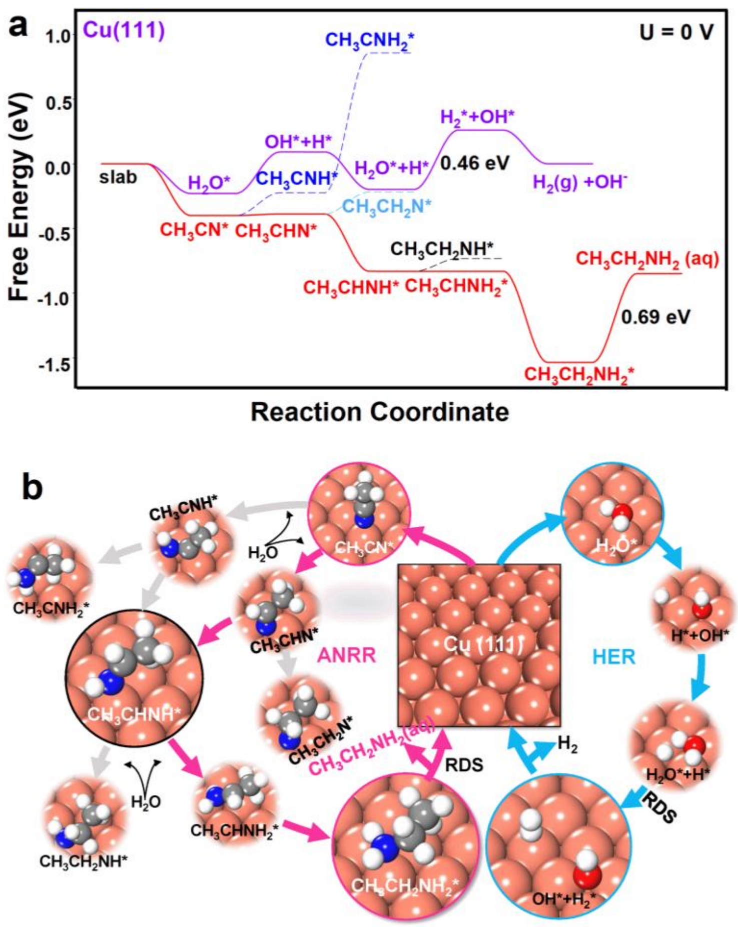

Supplementary Fig. 38 | The mechanism of ANRR on Cu(111) surface. a, Potential energy diagram of all possible pathways and **b**, corresponding geometric structures for the generation of EA by AN electroreduction on the Cu(111) surface at 0 V (vs RHE). The red line (**a**) and red arrows (**b**) denote the optimal path and the corresponding constructions; purple line (**a**) and blue arrow (**b**) denote the HER process.

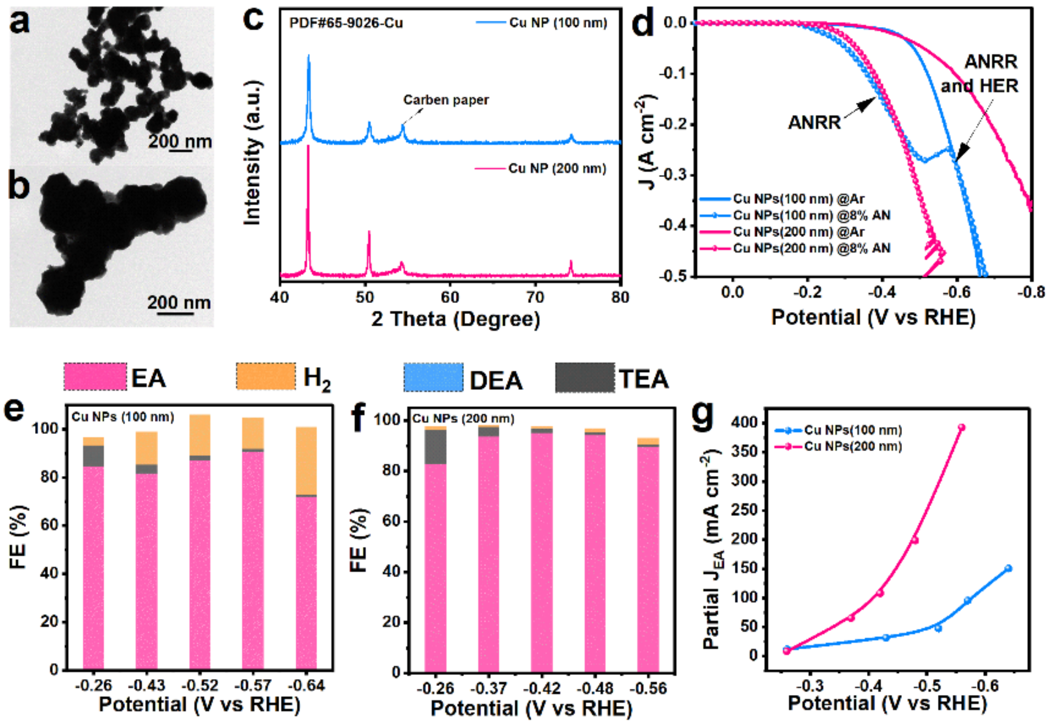

Supplementary Fig. 39 | Characterizations and catalytic performances of Cu NP (100 nm) Cu NP (200 nm). TEM images of **a**, Cu NP (100 nm) and **b**, Cu NP (200 nm). **c,** XRD patterns of Cu NP (100 nm) and Cu NP (200 nm). **d,** Linear sweep voltammetry (LSV) plots of Cu NP (100 nm) and Cu NP (200 nm) in argon-saturated 1 M KOH aqueous solutions with and without the addition of acetonitrile (AN) (8 wt%). Scan rate: 10 mV s^{-1}. Faradic efficiencies (FE) of various reduction products at different potentials on **e**, Cu NP (100 nm) and **f**, Cu NP (200 nm). **g,** Partial current densities of ethylamine at different potentials on Cu NP (100 nm) and Cu NP (200 nm). The a.u. stands for arbitrary units.

Experimentally, considering nanoparticles with larger size are generally supposed to possess higher coordination number (CN) and the smaller one possesses lower CN, we have conducted a comparative analysis of two copper nano-particles with distinct particle sizes (Cu NP (200 nm) and Cu NP (100 nm)). TEM and XRD analyses show the average particle diameters of Cu NP (100 nm) and Cu NP (200 nm) are ~100 nm and ~200 nm, respectively (**Supplementary Fig. 39 a-c**). The catalytic performance of both nanoparticles for the acetonitrile reduction with the same catalyst loading are assessed. Over the investigated potential range, compared with Cu NP (200 nm), Cu NP (100 nm) delivers lower current density, less attractive Faradaic efficiency (FE) and unsatisfying partial current density for acetonitrile reduction to ethylamine, particularly in the high potential region (<-0.55 V vs RHE) (**Supplementary Fig. 39 d-g**), demonstrating limited catalytic performance on Cu NP (100 nm). Thus, it seems that Cu NPs with lower coordination number could even obstacle the acetonitrile hydrogenation, suggesting that the changed coordination number (from 10.1 of Cu NWs to 9.3 of OD-Cu NWs) might not be critical factor for the highly efficient acetonitrile hydrogenation on OD-Cu NWs. Therefore, the difference of the catalytic activity is supposed to originate from the existence of lattice oxygen residues.

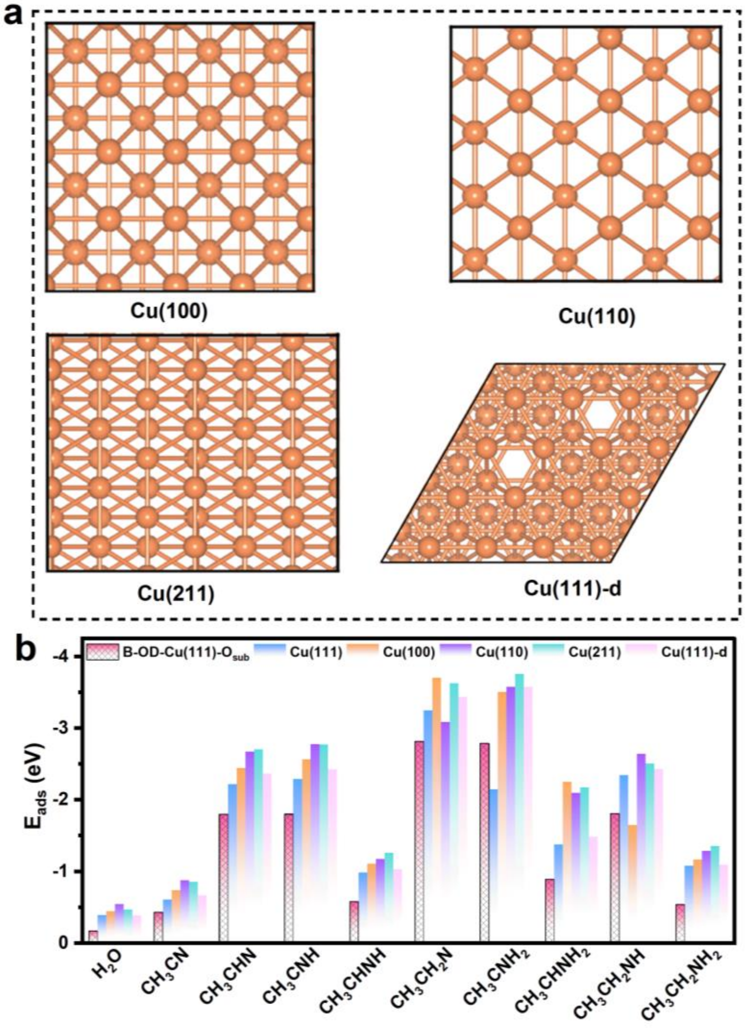

Supplementary Fig. 40 | DFT calculation the effect of coordination number (CN) on ANRR. a, Cu catalysts with various surface and bulk coordination configurations, including Cu(100), Cu(110), Cu(211), and Cu(111)-d. **b**, Comparison of the adsorption energies of critical reaction intermediates involved in the acetonitrile reduction reaction to form ethylamine among the Cu catalysts with different coordination configurations and oxygen residues.

The effects of unsaturated surface ligands (100, 211, 110) by constructing high index surfaces and unsaturated ligands (111-d) by creating copper vacancies under the surface on the reduction of acetonitrile were theoretically investigated (**Supplementary Fig. 35 a**). The results show that both unsaturated surface coordination and unsaturated bulk coordination exhibit stronger adsorption of amine intermediates (CH_3CN*, CH_3CHN*, CH_3CNH*, CH_3CHNH*, CH_3CH_2N*, CH_3CNH_2*, CH_3CH_2NH*, $CH_3CH_2NH_2$* and H_2O*) than the O-bearing B-OD-Cu surface, particularly for ethylamine adsorption, which is detrimental to the desorption of $CH_3CH_2NH_2$ from the catalyst surface and thus hinders the catalytic kinetics (**Supplementary Fig. 35 b**).

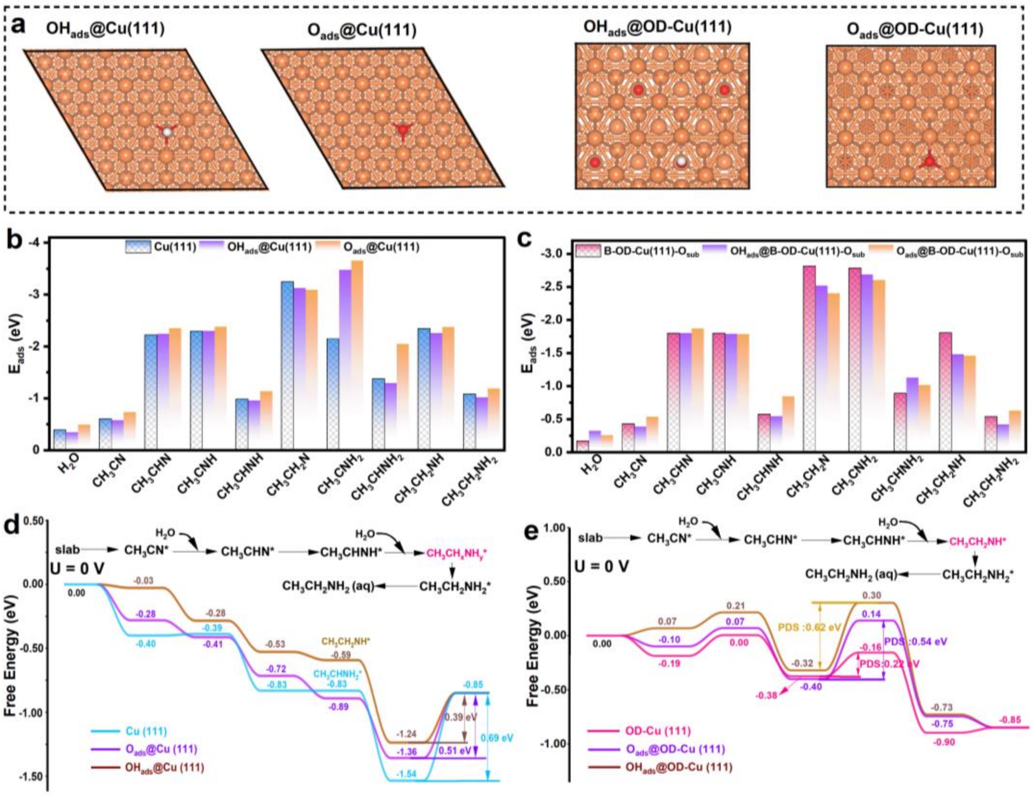

Supplementary Fig. 41 | DFT calculation the effect of dynamically adsorbed surface oxygen-containing species on ANRR. a, Configuration of Cu(111) and B-OD-Cu(111) catalysts adsorbing different oxygen-containing species (OH_{ads}@Cu(111), O_{ads}@Cu(111), OH_{ads}@OD-Cu(111) and O_{ads}@OD-Cu(111)). Adsorption energies of key intermediates involved in the acetonitrile reduction reaction on **b,** Cu and **c,** OD-Cu catalysts adsorbed by different oxygen-containing species. Potential energy diagrams of the optimal formation route for the electroreduction of acetonitrile to ethylamine over **d,** Cu and **e,** OD-Cu catalysts adsorbed with different oxygen-containing species at 0 V.

DFT calculations were also performed to investigate the effect of dynamically adsorbed surface oxygen-containing species on the electrochemical reduction of acetonitrile to ethylamine. **Supplementary Fig. 36 a-c** shows the configurations of Cu (111) and B-OD-Cu (111) surfaces with OH and O adsorption, and the adsorption energies of all intermediates involved in the electroreduction of acetonitrile to ethylamine. The results show that the adsorptions of amine intermediates on these surfaces with oxygen-containing species adsorption are slightly enhanced compared with pristine Cu (111) and B-OD-Cu (111) surfaces. The effect of surface-adsorbed oxygen-containing species on the reaction mechanism of the hydrogenation of acetonitrile to ethylamine is investigated. On Cu(111) surface, after introducing O* or OH*, the desorption of $CH_3CH_2NH_2$* remains as the potential determining step (PDS), which is similar to the pristine Cu(111). In comparison, on B-OD-Cu (111) surface, after adsorbing O* or OH*, the proton-coupled electron transfer electrochemical processes of CH_3CHNH* to CH_3CH_2NH* is still the PDS, consistent with B-OD-Cu (111) surface. Meanwhile, the energy barriers on Cu(111) surface and its oxygen-containing surfaces (O_{ads}@Cu(111) and OH_{ads}@Cu(111)) are always higher than on B-OD-Cu(111), O_{ads}@B-OD-Cu(111) and OH_{ads}@B-OD-Cu(111) surfaces (**Supplementary Fig. 36 d,e**). In a word, the dynamically formed oxygen-containing species would not change the PDS on either surfaces and would not change the trend of the energy barrier. Instead, the local surface electron distribution caused by lattice oxygen residues is believed to be the key factor to enhance the electroreduction of acetonitrile to ethylamine on the Cu metal surface.

Supplemental References

1 Branca, C. *et al.* Role of the OH and NH vibrational groups in polysaccharide-nanocomposite interactions: A FTIR-ATR study on chitosan and chitosan/clay films. *Polymer* **99,** 614-622 (2016).
